# Prediction and Quantification of Splice Events from RNA-Seq Data

**DOI:** 10.1371/journal.pone.0156132

**Published:** 2016-05-24

**Authors:** Leonard D. Goldstein, Yi Cao, Gregoire Pau, Michael Lawrence, Thomas D. Wu, Somasekar Seshagiri, Robert Gentleman

**Affiliations:** 1 Department of Bioinformatics and Computational Biology, Genentech Inc., South San Francisco, CA, United States of America; 2 Department of Molecular Biology, Genentech Inc., South San Francisco, CA, United States of America; University of California, Los Angeles, UNITED STATES

## Abstract

Analysis of splice variants from short read RNA-seq data remains a challenging problem. Here we present a novel method for the genome-guided prediction and quantification of splice events from RNA-seq data, which enables the analysis of unannotated and complex splice events. Splice junctions and exons are predicted from reads mapped to a reference genome and are assembled into a genome-wide splice graph. Splice events are identified recursively from the graph and are quantified locally based on reads extending across the start or end of each splice variant. We assess prediction accuracy based on simulated and real RNA-seq data, and illustrate how different read aligners (GSNAP, HISAT2, STAR, TopHat2) affect prediction results. We validate our approach for quantification based on simulated data, and compare local estimates of relative splice variant usage with those from other methods (MISO, Cufflinks) based on simulated and real RNA-seq data. In a proof-of-concept study of splice variants in 16 normal human tissues (Illumina Body Map 2.0) we identify 249 internal exons that belong to known genes but are not related to annotated exons. Using independent RNA samples from 14 matched normal human tissues, we validate 9/9 of these exons by RT-PCR and 216/249 by paired-end RNA-seq (2 x 250 bp). These results indicate that *de novo* prediction of splice variants remains beneficial even in well-studied systems. An implementation of our method is freely available as an R/Bioconductor package SGSeq.

## Introduction

More than 90% of genes in the human genome have multiple transcript isoforms [1, 2]. Transcript isoforms can be generated by alternative splicing of the primary mRNA transcript, transcription from alternative promoters, and cleavage at alternative 3′ polyadenylation sites [3]. Transcript variants can lead to protein isoforms with distinct function, changed UTRs with altered regulatory potential, or nonfunctional transcripts that are subject to nonsense-mediated decay (NMD). Alternative splicing plays an important role during development and in human diseases [4]. Moreover, genetic variation in the human population can affect the expression of individual transcript isoforms, and differential isoform usage may thus contribute to phenotypic diversity [5, 6].

High-throughput sequencing of RNA (RNA-seq) has replaced microarrays for gene expression studies and has enabled global analyses of transcript isoform expression. In an RNA-seq experiment, millions of short nucleotide sequence reads are generated from the ends of fragments in a cDNA library. The resulting reads are distributed along the length of the transcripts present in the sample, providing a comprehensive view of the selected transcriptome. Thus RNA-seq is well suited for the study of both known and novel transcript isoforms.

Most available methods for the genome-guided analysis of transcript variants from RNA-seq data fall into two categories: (1) Methods for the quantification of defined splice events (e.g. inclusion or skipping of a cassette exon), and (2) methods for the reconstruction and quantification of full-length transcripts. A detailed review of available tools can be found in [7].

**Fig 1:**
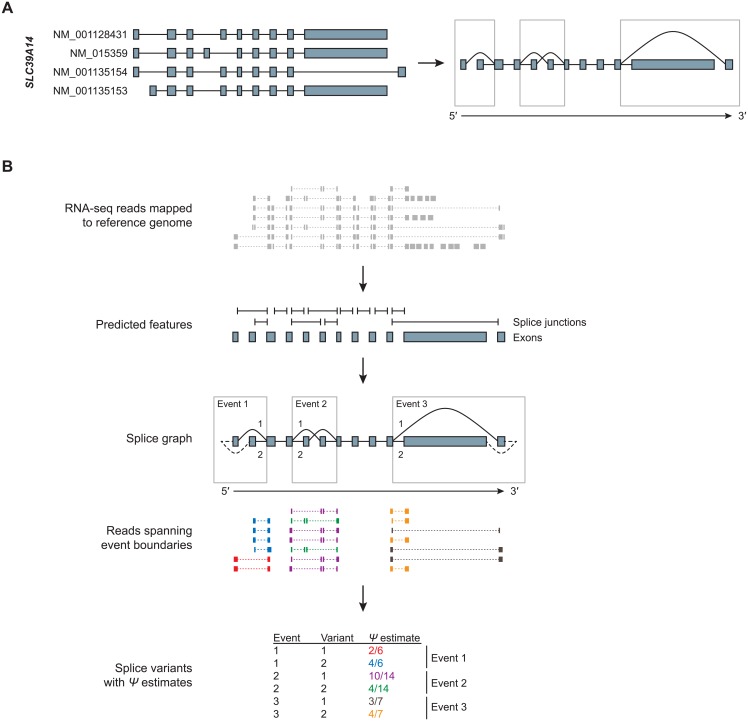
Splice graph and analysis workflow. A) Splice graph derived from four annotated transcript isoforms for gene *SLC39A14*. B) Schematic of analysis workflow. Discrete transcript features (splice junctions and exons) are predicted from RNA-seq reads mapped to a reference genome and are assembled into a splice graph. Splice events, characterized by two or more splice variants, are identified from the graph and estimates for relative variant usage Ψ are obtained based on reads spanning event boundaries.

Early studies of splicing based on RNA-seq data focused on the analysis of splice events [1]. Typically such studies consider a defined set of commonly occurring events, such as skipping or inclusion of a single cassette exon, pairs of exons that are included in transcripts in a mutually exclusive manner, exon extension or shortening due to alternative 5′ or 3′ splice sites, and alternative first or last exons. More generally the splice events of a gene can be described by its splice graph [8], a directed acyclic graph with nodes corresponding to transcript starts, ends and splice sites, and edges corresponding to exonic regions and splice junctions, directed from the 5′ to the 3′ end. When full-length isoforms are known, the splice graph can be constructed based on this information (Fig 1A). In the splice graph, regions shared between multiple isoforms are collapsed into a single path, while variant regions are represented by two or more paths belonging to distinct isoforms. Although the splice graph does not describe full-length isoforms, it describes all variant regions, and it has the advantage that sequence reads can be assigned unambiguously to a unique position in the graph. Available methods for analyzing splice events, such as MISO [9] or MATS [10], rely on annotation, and analyses are typically restricted to common types of splice events.

In contrast to event-centric methods, transcript reconstruction methods attempt to predict and quantify full-length transcripts. In this context, the short length of RNA-seq reads and the high similarity between isoforms pose technical challenges. Since most reads cannot be unambiguously assigned to individual transcripts, quantification is indirect and requires probabilistic models. Furthermore, transcript prediction and quantification often lead to identifiability issues. This is particularly problematic for transcript reconstruction, where in all but the simplest cases more than one set of transcripts can explain the observed data. To make the task mathematically tractable, methods rely on assumptions. For example, Cufflinks relies on parsimony, predicting a minimal set of transcripts consistent with the observed data [11]. Such assumptions may not always be appropriate and can lead to inaccurate predictions. Indeed an evaluation of 14 independent transcript reconstruction methods conducted by the RGASP consortium concluded that such methods yield varying results and do not perform well in complex organisms such as human [12].

Since most available event-centric methods do not perform prediction, hybrid approaches have been employed, relying on full-length transcript reconstruction for prediction, followed by an analysis of splice events based on predicted transcripts [13]. However, such approaches can be impractical when analyzing large data sets. Furthermore, calculating relative splice variant usage based on transcript-level abundances can lead to inaccurate quantification of splice events that could otherwise be quantified reliably from short read data.

To address the limitations of current approaches, we developed a novel method for the genome-guided prediction and quantification of splice events from RNA-seq data, which is implemented as an R/Bioconductor package SGSeq. We assess prediction accuracy based on simulated and real RNA-seq data, and illustrate how four different read aligners affect prediction results. We validate our approach for quantification based on simulated and real RNA-seq data, and compare local estimates of relative splice variant usage with those from two published methods. Furthermore, we demonstrate the utility of our approach by analyzing splice variants in normal human tissues, predicting and validating novel splice variants in known genes.

## Results

### Computational method

An overview of our method is shown in Fig 1B. For the annotation-free analysis of splice events, we predict exons and splice junctions from RNA-seq reads previously mapped to a reference genome, and assemble them into a genome-wide splice graph. Splice events are identified from the graph and are quantified locally based on reads that extend across the start or end of each splice variant.

**Fig 2:**
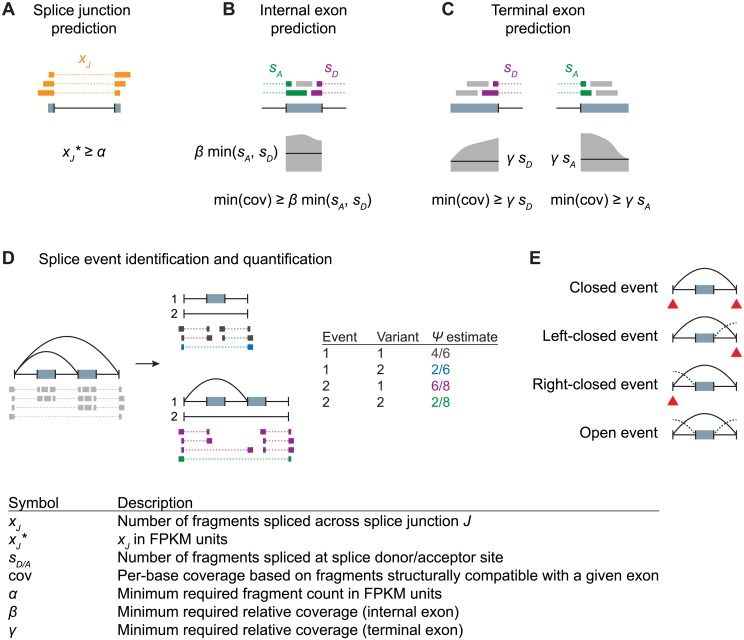
Transcript feature prediction and quantification of splice events. A) Splice junctions implied by split read alignments are filtered based on read counts in FPKM units. B) Genomic regions flanked by identified introns are predicted as internal exons if the minimum compatible coverage is at least *β* times the number of reads supporting the spliced exon boundaries. C) Genomic regions characterized by a flanking intron on one side and drop of coverage on the other side are predicted as terminal exons, with exon coordinates determined by the maximal region with compatible coverage at least *γ* times the number of reads supporting the spliced exon boundary. D) Nested events are identified recursively and quantified using reads spanning event boundaries. E) Schematic of exon skipping events that are closed, left-closed, right-closed or open. Informative event boundaries for which representative counts can be obtained are indicated with red triangles.

Initially splice junctions are extracted from split read alignments. The identified splice junctions are filtered based on their normalized count in FPKM units, controlled by parameter *α* (Fig 2A). Genomic regions flanked by a splice acceptor and a splice donor are candidate internal exons and are filtered based on their coverage with structurally compatible reads, controlled by parameter *β* (Fig 2B). Genomic regions characterized by an intron on one side and a drop of coverage on the other side are predicted as terminal exons, controlled by parameter *γ* (Fig 2C). For the analyses described in this study, predicted terminal exons sharing a splice donor or acceptor with a predicted internal exon are excluded. Prediction is performed on a per-sample basis to ensure the approach is scalable to large data sets. For data sets with multiple samples, per-sample predictions are subsequently merged to arrive at a common set of features that are used for further analysis.

Once exons have been identified based on structurally compatible reads, overlapping exons are split into disjoint exon bins. The resulting exon bins, together with the identified splice junctions, define a genome-wide splice graph from which splice events can be identified. In our framework, a splice event is characterized by a start node and an end node that are connected by at least two paths, and there is no intervening node with all paths intersecting. To account for events involving alternative transcript starts and ends, the subgraph for each gene is extended by adding a unique *source* node connected to all transcript start nodes and a unique *sink* node reachable from all transcript end nodes. Once events are identified in terms of their start and end, alternative paths (or splice variants) can be described recursively (Fig 2D).

Prediction and quantification are performed in separate steps. For a set of features (predicted or extracted from annotated transcripts) counts of compatible reads are obtained for each sample, resulting in a rectangular matrix of counts. For the quantification of splice variants, we are interested in estimating the relative usage, or variant frequency, denoted by Ψ. We obtain counts for each variant based on structurally compatible reads extending across the start or end of the variant (Fig 2D). Depending on the type of splice event, representative reads can be junction reads as shown in Figs 1 and 2, or they can be unspliced reads that overlap adjacent disjoint exon bins. Dividing the count for a variant by the total count for all variants belonging to the same event yields a local estimate for relative usage at the start and end of the variant. Local estimates are then combined using a weighted mean, to obtain a single estimate per variant (see Methods).

A complication arises in the case of overlapping events (Fig 2E). We say an event is *left-closed* if a node that is part of the event can be reached from an external node through the event start only. Similarly, we say an event is *right-closed* if all paths from nodes inside the event to outside nodes must pass through the event end. For quantification of splice variants, counts at the event start and end are valid only if the event is right-closed and left-closed, respectively.

Our approach is implemented as an open-source R/Bioconductor package SGSeq, which is freely available from the Bioconductor project website (www.bioconductor.org) [14].

### Prediction accuracy

To assess the performance of our algorithm in terms of prediction accuracy, we obtained ENCODE RNA-seq data (89.5M read pairs) from a cell line HepG2 previously used for an assessment of transcript reconstruction methods [12]. Our approach takes as input reads mapped to a reference genome and is thus sensitive to the performance of the read alignment program, in particular its ability to accurately map reads across exon-exon junctions without prior knowledge of the underlying splice event [15]. We therefore considered four different alignment methods for our benchmarking study. We mapped reads to the human reference genome using GSNAP [16] (61.3M unique and concordant read pairs), HISAT2 [17] (57.8M properly aligned read pairs), STAR [18] (72.2M properly aligned read pairs), and TopHat2 [19] (33.3M properly aligned read pairs). Prediction accuracy was evaluated in terms of false discovery rate (FDR) with respect to known features in the refGene, knownGene, GENCODE (v19) and lincRNA tables [20, 21]. Predicted splice junctions and internal exons were considered true if they are identical to splice junctions or internal exons in an annotated transcript. Due to variability in read coverage, precise prediction of transcript starts and ends from RNA-seq data is problematic [12]. We therefore used more relaxed criteria when assessing terminal exon predictions, considering a predicted terminal exon true if its spliced boundary agrees with that of an annotated terminal exon.

**Fig 3:**
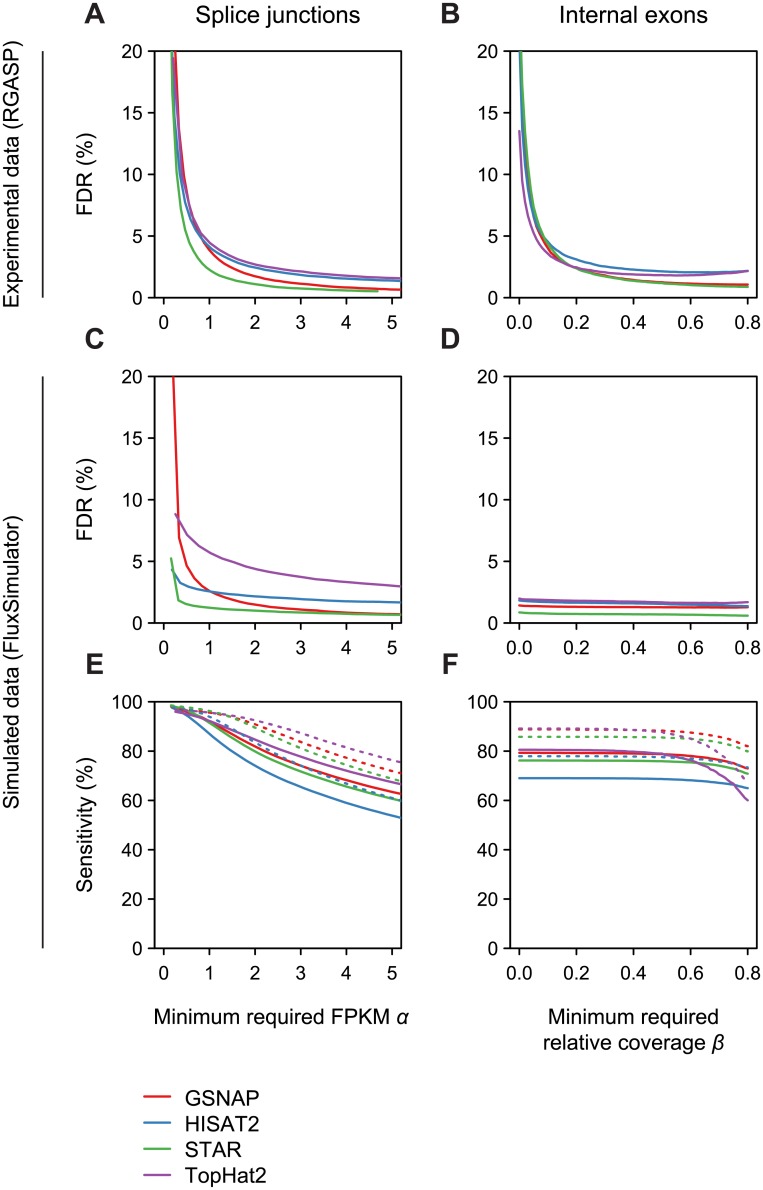
Prediction accuracy. A,B) Analysis of false discovery rate (FDR) based on real RNA-seq data, assessing (A) the effect of minimum required FPKM (*α*) for splice junction prediction, and (B) the effect of minimum required relative coverage (*β*) for internal exon prediction. C,D) Analysis of FDR based on simulated RNA-seq data, otherwise as in (A, B). E,F) Analysis of sensitivity based on simulated RNA-seq data, for prediction of splice junctions (E) and internal exons (F). Solid and dashed lines in (E,F) correspond to values obtained with transcripts expressed at FPKM >1 and 2, respectively. Colors correspond to different read mapping programs as indicated in the color key.

First we assessed FDR for splice junction prediction. Since some splice junctions observed in aligned reads are the result of spurious alignments, additional filtering is required to control FDR. This can be achieved by setting a minimum required FPKM using parameter *α* (Fig 3A). Without filtering (*α* = 0) FDR is high (ranging between 19–59%, depending on the aligner) but decreases rapidly for increasing *α*. For *α* = 2 the estimated FDR ranged between 1.1–2.7%, depending on the alignment method. In the ENCODE data, a minimum FPKM of 2 corresponded to a minimum read count of 19, 18, 22, 10 for GSNAP, HISAT2, STAR and TopHat2 alignments, respectively. Beyond FDR control, parameter *α* specifies the minimum expression level for splice junctions of interest, and appropriate values therefore depend on the aims of a particular analysis. Next we assessed the prediction of internal exons. Fig 3B illustrates the effect of varying *β*, specifying the minimum required coverage relative to the number of reads spliced at the exon boundaries. For *α* = 2 and *β* = 0.2 the estimated FDR ranged between 2.4–3.1%.

A limitation of the described benchmarking analysis, comparing predictions from real RNA-seq data with annotated transcript features, is the fact that truly expressed transcripts are unknown, and our set of annotated transcripts may be incomplete. We therefore used a complementary benchmarking approach, by simulating 50 million RNA-seq read pairs from refGene transcripts, using the FluxSimulator software [22]. We mapped simulated reads to the human reference genome with the four alignment programs as previously, obtaining 39.8M, 37.0M, 41.6M and 26.1M mapped read pairs with GSNAP, HISAT2, STAR and TopHat2, respectively. Results for splice junction predictions were mostly consistent with results from real RNA-seq data (Fig 3C). For *α* = 2, FDR estimates ranged between 1.0–2.1% for GSNAP, HISAT2 and STAR. However, FDR estimates for internal exon prediction were uniformly low, even for small values of *β*, indicating a limitation of simulated data (Fig 3D). In real RNA-seq data, reads can derive from introns of unspliced pre-mRNAs, and requiring sufficient read coverage for exon prediction ensures that such reads do not adversely affect predictions. However, in well-behaved simulated data lacking intronic reads, the effect of *β* on prediction results cannot be properly assessed. The simulated data also allowed us to assess prediction sensitivity. For *α* = 2, sensitivity estimates for splice junction predictions ranged between 72–84% and 82–92% for transcripts expressed at FPKM >1 and FPKM >2, respectively (Fig 3E). For internal exon predictions with *α* = 2 and *β* = 0.2, sensitivity estimates ranged between 69–80% and 78–89% for transcripts expressed at FPKM >1 and FPKM >2, respectively (Fig 3F).

We observed that terminal exons were predicted with reasonable sensitivity (68–80% and 79–88% for 5′- and 3′-terminal exons for simulated reads from transcripts with FPKM >2, respectively) but poor precision (FDR based on simulated data 35–44% and 30–37%, respectively) (data not shown). The high FDR in this case was due to internal exons with low read coverage being predicted as terminal exons. Indeed, when accepting a predicted 5′-(3′-)terminal exon as true if it shared a splice donor (acceptor) with either a terminal or internal expressed exon, FDR dropped to 3.4–6.3% and 3.4–7.9% for 5′- and 3′-terminal exons, respectively. This indicated that our approach was often unable to reconstruct the full splice graph of a gene. However, it remains possible to identify and quantify splice events with sufficient read coverage in the context of an incomplete splice graph.

Finally, parameter *γ* determines the unspliced boundary of predicted terminal exons, by specifying a minimum read coverage relative to the number of spliced reads at the spliced exon boundary. An assessment of sensitivity and precision at the base-level indicated that a required relative coverage of *γ* = 0.2 achieved a good balance between sensitivity and precision (S1 Fig).

### Splice variant quantification with SGSeq, MISO and Cufflinks

**Fig 4:**
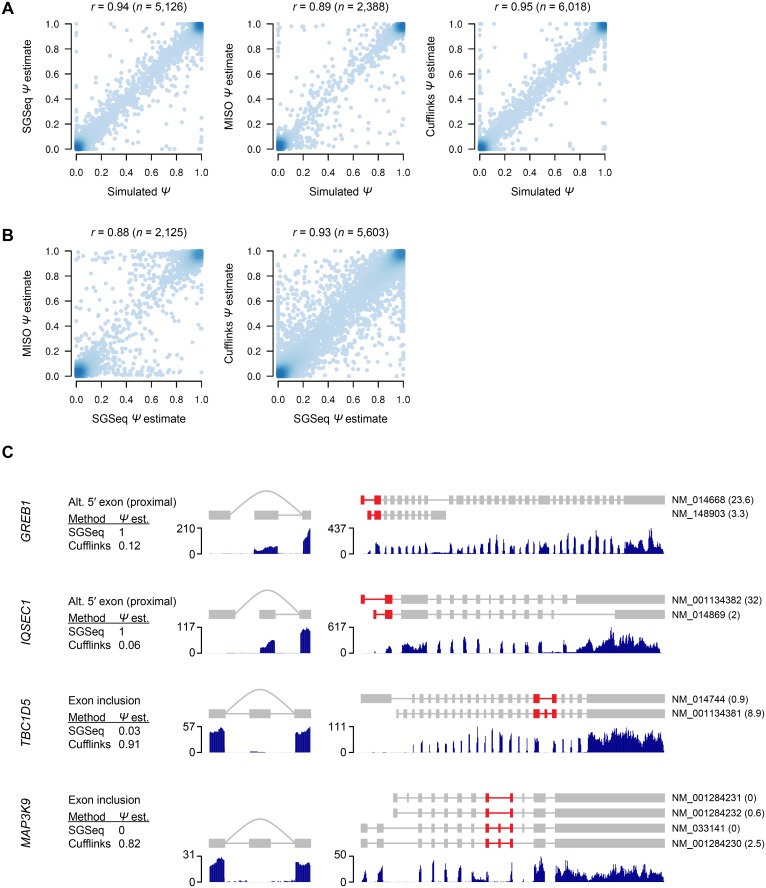
Splice variant quantification with SGSeq, MISO and Cufflinks. A) Comparison of estimates of relative splice variant usage Ψ obtained by SGSeq, MISO and Cufflinks with true Ψ values underlying simulated data. B) Comparison of estimates of relative splice variant usage Ψ obtained by SGSeq, MISO and Cufflinks based on real RNA-seq data. C) Comparison of Ψ estimates obtained with SGSeq and Ψ estimates inferred from Cufflinks transcript-level estimates. Splice events (left) are highlighted in the context of full-length transcripts (right). Introns are not drawn to scale. RNA-seq read coverage is shown below events and transcripts. Results are based on GSNAP alignments. Cufflinks Ψ estimates were inferred from transcript-level expression estimates.

Next we assessed the accuracy of estimates for relative splice variant usage. For this purpose, we obtained splice events implied by refGene transcripts (see Methods) and determined their relative usage in our simulated data. We obtained Ψ estimates with SGSeq, as well as two previously published methods, MISO [9] and Cufflinks [11]. MISO estimates Ψ using a Bayesian framework, while Cufflinks estimates expression levels for full-length transcripts and does not estimate Ψ directly. Since the abundance of a splice variant is equal to the total abundance of transcripts containing the variant, it is possible to infer Ψ estimates from transcript-level estimates. For Cufflinks, we calculated Ψ estimates as the sum of expression levels for transcripts containing the variant, divided by the sum of expression levels for transcripts that include variants belonging to the same event. Fig 4A illustrates that all three methods arrive at estimates that, in most cases, closely match simulated values (SGSeq *r* = 0.94, MISO *r* = 0.89, Cufflinks *r* = 0.95, Spearman correlation coefficient). Similar results were obtained with different alignment methods (S2 Fig).

Although all three methods were able to quantify relative splice variant usage for simulated data, it is unclear how they perform when applied to real RNA-seq data, in particular when expressed transcripts are not in agreement with the annotation used for quantification. We therefore also quantified refGene splice events in the ENCODE cell line data, and compared Ψ estimates for the three different methods. In most cases SGSeq Ψ estimates agreed well with those obtained with MISO (*r* = 0.88) and Cufflinks (*r* = 0.93) (Fig 4B, S3 Fig). However, for some events SGSeq and Cufflinks differed substantially (Fig 4B), and we asked whether discrepancies in these cases are due to event-centric compared to transcript-centric quantification.

Inspection of mapped reads at individual events confirmed that incomplete or incorrect transcript models can have adverse effects on quantification. For example, Fig 4C illustrates alternative first exons in *GREB1* and *IQSEC1*, where transcription appears to be almost exclusively from the downstream exon (SGSeq $\hat{\Psi }=1$ in both cases). However, since transcripts including the upstream exon are in closer agreement with the RNA-seq data for other parts of the transcript, Cufflinks estimates yield Ψ estimates of 0.12 for *GREB1* and 0.06 for *IQSEC1*. Similarly, two cassette exons in *MAP3K9* and *TBC1D5* appear to be absent or expressed at low relative levels compared to flanking exons (SGSeq $\hat{\Psi }=0$ and $\hat{\Psi }=0.03$, respectively). However, refGene transcripts supporting inclusion or skipping of the cassette exons differ in other parts of the transcripts, affecting their quantification. For example, in the case of *MAP3K9*, all transcripts including the cassette exon are transcribed from an upstream transcript start site (TSS), while all transcripts skipping the cassette exon are transcribed from a downstream TSS. In the test data, *MAP3K9* appears to be transcribed exclusively from the upstream TSS, and thus Cufflinks yields higher estimates for transcripts including the cassette exon.

### Identification of novel exons in normal human tissues

Annotation of the human transcriptome is continuously improving and becoming more complete [23]. We therefore asked whether there is benefit in predicting gene models from RNA-seq data, compared to the use of existing annotation. For a proof-of-concept study we obtained paired-end RNA-seq data from 16 normal human tissues (Illumina Human Body Map 2.0) and used SGSeq to perform a genome-wide prediction of transcript features (Table 1).

**Table 1:** Summary of transcript features predicted from 16 paired-end RNA-seq samples (Illumina Body Map 2.0). Splice junctions and internal exons were considered annotated if they are identical to annotated splice junctions or internal exons, respectively. 5′/3′-terminal exons were considered annotated if they share a splice donor/acceptor with an annotated terminal or internal exon. Reported exons are predicted exons, prior to disjoining them into disjoint exon bins.

Feature type	Predicted	Annotated	% Annotated
Splice junction	192,288	178,320	92.7%
Internal exon	139,691	134,735	96.5%
5′ exon	39,588	35,089	88.6%
3′ exon	37,263	32,928	88.4%

Predictions define a genome-wide splice graph for transcribed genomic loci, which include both known and unannotated genes. For the purpose of this study, we focused on candidate novel exons of known genes. In particular we performed a detailed analysis of predicted internal exons that belong to known genes, but do not overlap any annotated exons (*n* = 249, S1 Table).

**Fig 5:**
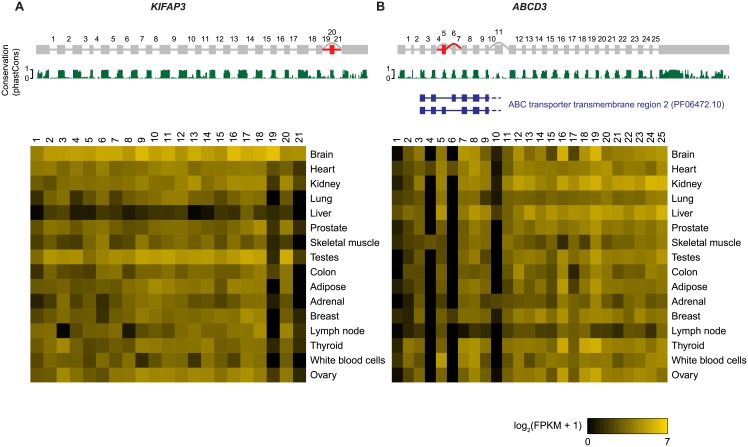
Predicted splice graph and splice junction expression for *KIFAP3* and *ABCD3*. Heatmaps are based on splice junction expression on a log_2_(FPKM + 1) scale. Grey and red indicate annotated and unannotated transcript features, respectively. Introns are not drawn to scale. PhastCons scores indicating evolutionary conservation are shown in green below each splice graph. The position of Pfam domain ‘ABC transporter transmembrane region 2’ (PF06472.10) is indicated in blue below the splice graph for *ABCD3*.

Genes with candidate novel exons include several with known biological function. Fig 5 shows the predicted splice graphs for the kinesin-associated protein *KIFAP3* and the ATP-binding cassette transporter gene *ABCD3*, together with expression of individual splice junctions across normal human tissues. Predictions indicate a novel brain-specific cassette exon in *KIFAP3*, as well as a novel exon in *ABCD3* expressed in heart and skeletal muscle. The novel exons show evidence for evolutionary conservation comparable to annotated exons (Fig 5) [24].

### Validation of predicted novel exons

**Fig 6:**
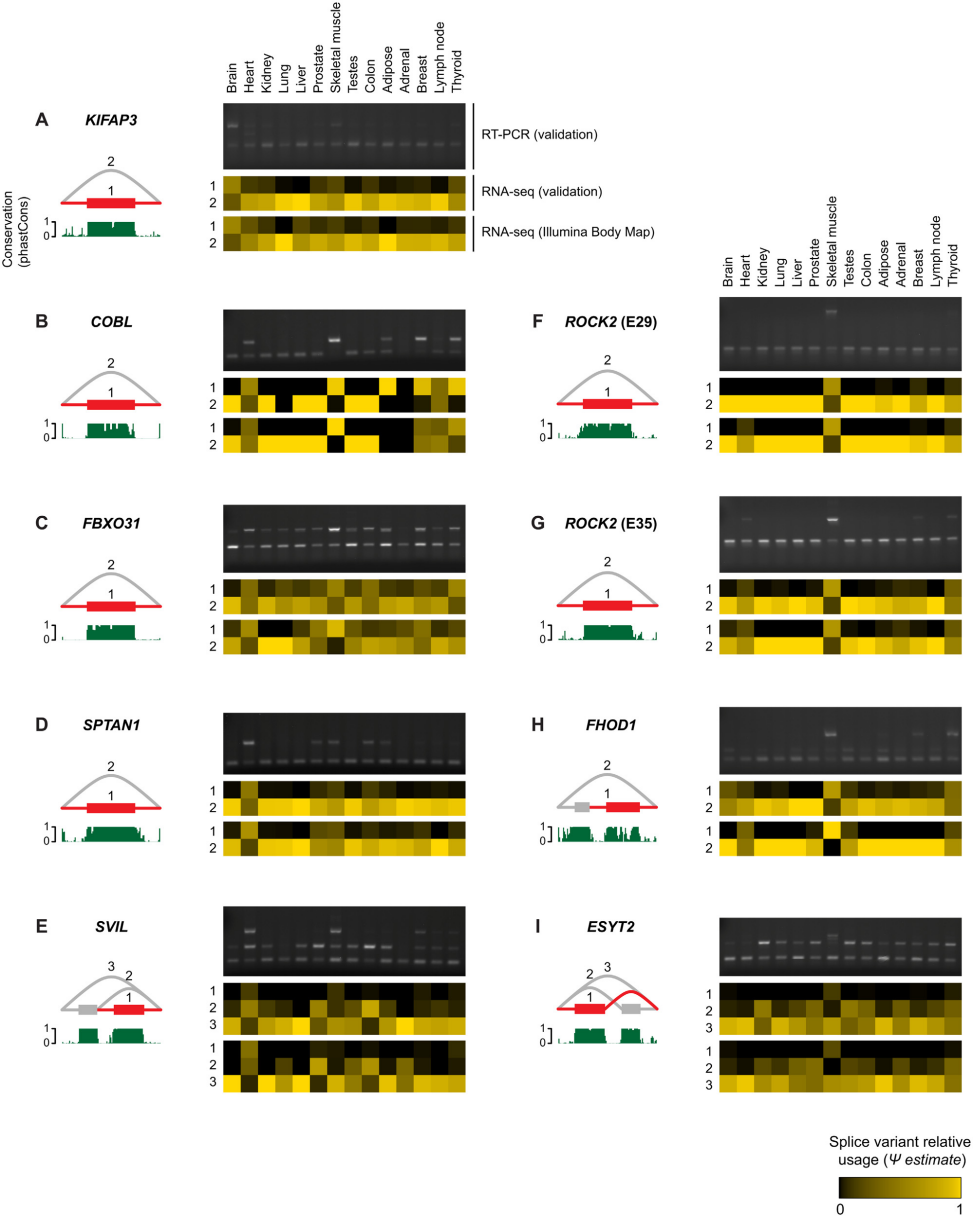
Estimates of relative usage Ψ for candidate novel exons across normal human tissues and validation by RT-PCR. A-I) Splice graph of predicted events. Grey and red indicate annotated and unannotated transcript features, respectively. Introns are not drawn to scale. PhastCons scores indicating evolutionary conservation are shown in green below each splice graph. Heatmaps illustrate estimates for variant frequency Ψ. In each panel, heatmaps for 14 tissues are based on RNA-seq data from the Illumina Body Map (bottom) and validation samples (top). RT-PCR results were obtained with primers targeting flanking exons.

We selected nine candidate novel exons in eight different genes for validation by RT-PCR in independent samples for 14 out of 16 tissues that were included in the RNA-seq data. The candidates include simple cassette exons, but also exons that are part of more complex splice events. We designed primers against flanking constitutive exons, such that splice variants can be distinguished based on the size of the PCR product. RT-PCR experiments verified the existence of all nine predicted exons, and the RT-PCR data showed tissue-specific expression similar to the RNA-seq data (Fig 6).

For *KIFAP3* and *FHOD1* (Fig 6A and 6H) RT-PCR results suggested the presence of additional splice variants. We therefore asked whether our initial genome-wide analysis was not sensitive enough in these cases. Indeed, when re-analyzing the relevant loci with higher sensitivity, additional splice variants could be identified and quantified in agreement with RT-PCR results (S4 Fig).

For a more global validation of predicted exons, we performed paired-end RNA-seq experiments (2 x 250 bp) on the 14 samples previously used for RT-PCR. When using the newly generated RNA-seq data to quantify the nine splice events described previously, we observed close agreement with the Illumina Body Map data and with the RT-PCR experiments, as expected (Fig 6). To validate additional predicted candidate exons, we required an exon to be fully sequenced. Specifically, we considered a predicted exon validated, if a single pair of reads covered the exon in its entirety (without gaps) and was split at the exon start and end, thus lending support to its spliced boundaries. Using these criteria we were able to validate 216/249 (87%) of the predicted candidate exons (S1 Table).

### Potential function of novel coding exons

Among 249 candidate exons, 231 are spliced into protein-coding transcripts, and 198 are predicted to affect coding sequences (S1 Table). The same exon can have different effects on coding potential, depending on the full-length transcript. Seventy-seven exons were predicted to cause in-frame alterations for all annotated transcript isoforms, while 92 exons were predicted to result in either a premature stop codon or a frame-shift with altered stop codon for all annotated transcript isoforms. Stop codons can result in transcript degradation through the NMD pathway if the stop codon is more than ∼50 nucleotides upstream of the 3′-most exon-exon junction [25]. In 31 of 92 cases that involved stop codon alterations, the new stop codon was situated within 55 nucleotides of the 3′-most exon-exon junction for some transcripts, suggesting that these transcripts may escape NMD. This category includes the novel exon in *KIFAP3* shown in Fig 6A. Next we translated predicted in-frame exons in the context of their flanking exons, and scanned the amino acid sequences for known protein domains in the Pfam database. In two cases, novel exons appear to encode known Pfam domains (see Methods). In the case of *ABCD3*, a novel exon occurs mutually exclusively with an annotated exon. Both exons are highly evolutionary conserved, have identical length and both show homology to the same part of the ABC transporter transmembrane domain (Fig 5B), suggesting that preferential inclusion of the alternative exon in heart and skeletal muscle may confer a specialized function in these tissues.

## Discussion

In this study, we present a novel method for the genome-guided prediction and quantification of splice events from RNA-seq data. Previous studies allowed for unannotated splice junctions between known exons, but did not account for unannotated exons [2, 26]. A recently published method DiffSplice supports exon prediction, but was not available to us for testing due to licensing restrictions by the authors [27]. Here we demonstrate that splice junctions and internal exons with sufficient read coverage can be predicted with high precision and sensitivity, without relying on annotation.

Our approach does not attempt to predict the structure of full-length transcripts. Given the read lengths obtained with widely used RNA-seq technologies, full-length transcript prediction and quantification requires assumptions that may be inappropriate and can adversely affect results (Fig 4C). Our comparison of event-level and transcript-level quantification illustrates the limitations of transcript-centric methods and the advantages of local prediction and quantification. Specifically, local prediction avoids the problematic transcript reconstruction step, and it is more robust to incomplete or inaccurate transcript annotation.

Despite large-scale efforts towards a complete annotation of the human transcriptome [28], we were able to predict and validate unannotated exons expressed in normal human tissues. Some of the candidate novel exons are included in a tissue-specific manner and likely confer biological function. These findings illustrate that accounting for unannotated splice variants can lead to new discoveries even in well-studied systems. Of the 249 candidate novel exons, 72 were predicted in a previous analysis of the Illumina Body Map data using Cufflinks [13]. Exons not identified in the previous study included exons in *SVIL* and *ESYT2* validated by RT-PCR.

Our method enables the analysis of unannotated and complex splice events from short read RNA-seq data. We anticipate that it will be beneficial for the study of organisms with incomplete transcript annotation, as well as in situations where unannotated splice events are expected. We expect that it will be particularly useful for studies of genetic variation resulting in unannotated splice variants, as well as for the study of diseases that lead to or are caused by aberrant transcripts, such as human cancers.

## Materials and Methods

### RNA-seq read alignment

RNA-seq reads were mapped to the human reference genome (GRCh37/hg19). GSNAP (2013-10-10) [16] was run with parameters -M 2 -n 10 -B 2 -i 1 -N 1 -w 200000 -E 1 --pairmax-rna = 200000 --clip-overlap -a paired. Only unique and concordant alignments were used for downstream analysis. HISAT2 (2.0.0) [17] was run with option --dta-cufflinks. STAR (2.4.2a) [18] was run with option --outSAMstrandField intronMotif to generate the XS strand attribute for spliced alignments. TopHat2 (2.0.13) [19] was run with default parameters. Alignments from all programs were subsequently filtered with samtools [29] to retain only properly paired reads.

### Simulation

RNA-seq reads from refGene transcript were simulated with the FluxSimulator software (1.2.1) [22] using the configuration file for ‘RNA hydrolysis protocol’ with the following modifications: NB_MOLECULES 500000000, READ_NUMBER 100000000, READ_LENGTH 76, PAIRED_END YES, FASTA YES, ERR_FILE 76, UNIQUE_IDS YES.

### Normalized read counts

Disjoint exon bins and splice junctions are quantified using normalized read counts in FPKM units (fragments per kilobase and million) based on structurally compatible fragments. For single-end and paired-end data, fragment counts are equivalent to counts of reads and read pairs, respectively. FPKM was calculated as

$$x^{*}=x{\left(\lambda \rho \right)}^{-1}10^{9},$$

where *x* is the number of structurally compatible fragments, *ρ* is the library size (number of aligned fragments) and *λ* is the effective feature length defined as the number of possible positions for a compatible fragment. For single-end data

λ=l+r-1,

where *l* and *r* are feature length and read length, respectively (a splice junction has length *l* = 0).

In the paired-end case, effective length is given by

λ=l+f-1-max(i-l+1,0),

where *l* is as defined previously, and *f* and *i* are fragment length and distance between fragment ends *i* = *f* − 2*r*, respectively. The last term accounts for the case when *i* ≥ *l* and hence the alignment can overlap the feature without fragment ends overlapping it. Note that fragment length is generally unknown, since the genomic region between paired alignments can include introns that are absent from the transcript the fragment derived from. In the current implementation, we obtain a per-sample estimate of *f* using the median insert size for aligned read pairs.

### Quantification of splice events

Local estimates of relative splice variant usage are obtained at the start and/or end of each splice variant. Consider an event with *n* variants. For each variant *i* (*i* = 1, …, *n*) we obtain $x_{i}^{D}$, the number of compatible fragments with reads overlapping the splice donor at the start of the event, and $x_{i}^{A}$, the number of compatible fragments with reads overlapping the splice acceptor at the end of the event. Let $m^{S}=\sum_{k=1,\ldots ,n}x_{k}^{S}$ and calculate relative usage for variant *i* as ${\hat{\Psi }}_{i}^{S}=x_{i}^{S}/m^{S}$ for *S* = *D*, *A*. For events starting at a *source* or ending at a *sink* node (involving alternative transcript starts or ends), estimates are only obtained at the end and start of the event, respectively. For events with two valid local estimates, local estimates are combined using a weighted mean, giving higher weight to estimates that are based on higher counts

$${\hat{\Psi }}_{i}=\frac{m^{D}}{m^{D}+m^{A}}{\hat{\Psi }}_{i}^{D}+\frac{m^{A}}{m^{D}+m^{A}}{\hat{\Psi }}_{i}^{A}=\frac{x_{i}^{D}+x_{i}^{A}}{m^{D}+m^{A}}.$$

### Software implementation

Our method is implemented as an open-source R/Bioconductor package SGSeq and is freely available from the Bioconductor project website (http://www.bioconductor.org) [14]. We make extensive use of the genomic ranges infrastructure for sequence analysis [30] as well as the R package igraph [31].

### Benchmarking of quantification

We compared SGSeq (1.4.0) with MISO (0.5.2) [9] and Cufflinks (2.2.1) [11]. refGene transcripts were quantified using cuffquant and cuffnorm. Splice events were extracted from refGene annotation. Only closed binary events of type ‘skipped exon’, ‘alternative 5′ splice site’, ‘alternative 3′ splice site’, ‘alternative first exon’ and ‘alternative last exon’ were considered. We considered MISO estimates with informative reads ≥ 20 and confidence interval < 0.2, and SGSeq estimates with *m*^*D*^ + *m*^*A*^ ≥ 20.

### Analysis of Illumina Body Map data

Illumina Body Map paired-end RNA-seq data were processed by trimming poor quality 3′ bases, and removing poor quality reads, as well as ribosomal reads. Processed reads were aligned with GSNAP (2013-10-10) [16] as described above, except that known splices were provided using option -s (known splices were based on RefSeq transcripts, downloaded on 30 November 2011). Only unique and concordant alignments were considered. SGSeq was run with default parameters (*α* = 2, *β* = 0.2, *γ* = 0.2). Among predicted internal exons, we selected candidate novel exons in known genes. To simplify downstream analyses, we only considered internal exons with fixed start and end coordinates (no alternative splice sites) and unambiguous flanking splice junctions in our data set. Exons were required to (1) be connected to the splice graph of a known gene, (2) overlap with the annotated gene locus, (3) be spliced to annotated exons, and (4) not overlap any annotated exons.

### Analysis of protein domains

For exons that were predicted to cause in-frame alterations for all annotated protein-coding transcripts, we translated the altered full-length transcripts and obtained amino acid sequences of the predicted exons, in the context of 10 upstream and 10 downstream flanking residues. We used the hmmscan program in the HMMER (3.1b1) software package [32] to scan 77 amino acid sequences for protein domains in the Pfam database (Release 27.0) [33]. We retained domain hits with independent E-value × 77 < 0.2. This resulted in two hits for domains ‘ABC transporter transmembrane region 2’ (PF06472.10) in *ABCD3* and ‘Domain of unknown function (DUF1908)’ (PF08926.6) in *MAST3*.

### Normal human RNA samples

Total RNA from 14 normal human tissues were purchased from Ambion and BioChain. RNA samples are de-identified and hence are not considered human subject research under the US Department of Human and Health Services regulations and related guidance (45 CFR Part 46). According to the Ambion website, all human tissues used for RNA isolation are obtained through accredited organ/tissue procurement organizations that operate with Institutional Review Board approval, and in accordance with regulations set forth by the US Department of Health and Human Services. These regulations include donor consent and protection of privacy regulations. According to the BioChain website, BioChain complies with the relevant regulations from the various governing bodies when handling biomaterials and related patient information. This includes approval of the protocol by the Investigation Review Board, the informed consent form for donors or participants, confidentiality/privacy of related information, and quality assurance in the process.

### RT-PCR experiments

RT-PCR experiments were performed using random hexamer, superscript II and platinum Taq according to the manufacturer’s instructions (Invitrogen). The PCR products were visualized on 3% agarose gels.

### RNA-seq experiments

RNA-seq was carried out on total RNA from normal human tissue samples that were also subjected to RT-PCR. Libraries were prepared using the TruSeq RNA Sample Preparation Kit (Illumina) and sequenced on the Illumina HiSeq 2500 instrument. On average ∼70 million 2 x 250 bp paired reads were obtained for each sample.

## Supporting Information

S1 Fig Prediction accuracy for terminal exon boundaries. A,B) Analysis of false discovery rate (FDR) at the nucleotide level based on real RNA-seq data, assessing the effect of minimum required relative coverage (*γ*) on (A) 5′- and (B) 3′-terminal exons. C,D) Analysis of FDR at the nucleotide level based on simulated RNA-seq data, otherwise as in (A, B). E,F) Analysis of sensitivity at the nucleotide level, based on simulated RNA-seq data. Solid and dashed lines in (E,F) correspond to values obtained with transcripts expressed at FPKM >1 and 2, respectively. Colors indicate different alignment programs as described in the color key.
(EPS)

S2 Fig Splice variant quantification with SGSeq, MISO and Cufflinks for simulated data. Comparison of estimates for relative splice variant usage Ψ obtained by SGSeq, MISO, Cufflinks with true Ψ values underlying simulated data for different read alignment programs. Cufflinks Ψ estimates were inferred from transcript-level expression estimates.
(EPS)

S3 Fig Splice variant quantification with SGSeq, MISO and Cufflinks for real RNA-seq data. Comparison of estimates for relative splice variant usage Ψ obtained by SGSeq, MISO, Cufflinks based on real RNA-seq data for different read alignment programs. Cufflinks Ψ estimates were inferred from transcript-level expression estimates.
(EPS)

S4 Fig Analysis of *KIFAP3* and *FHOD1* with more sensitive prediction parameters. Re-analysis of splice events in *KIFAP3* (A) and *FHOD1* (B) based on predictions with high sensitivity (*α* = 0.5, *β* = 0.2, *γ* = 0.2). Otherwise as in Fig 6.
(EPS)

S1 Table Candidate novel exons in known genes. Candidate novel exons in known genes predicted from Illumina Body Map RNA-seq data (see Methods).
(XLS)
